# A classification of foot types of high school girls in South Korea

**DOI:** 10.1186/1757-1146-7-S1-A106

**Published:** 2014-04-08

**Authors:** Saemi Shin, Jongsuk Chun

**Affiliations:** 1 Symbiotic Life Tech., Yonsei University, Seodaemun-gu, Seoul, 120-749, Korea; 2 Dept. of Clothing and Textiles, Yonsei University, Seodaemun-gu, Seoul, 120-749, Korea

## 

Wearing uncomfortable shoes for a long time may cause pains by blisters or corns in the foot. It may lead to weaken ligaments and muscles of lower limbs and feet. The previous researchers claimed that a pain in the foot may be prone to a backache [1]. High school girls feel discomfort in their foot for wearing shoes, especially the heel, toes, or sole, caused by the shape, height of heel, or ball girth of the shoe [2]. The aim of this study was to analyze characteristics according to the forms of the foot that would be applied to make well-fitted shoes for female South Korean youths. This study classified foot types of South Korean high school girls of 17 and 18 years. The three-dimensional foot scan data were collected with a 3D foot scanner. 201 subjects were participated in the experiment. Twenty-four dimensions were measured on the right foot: 6 lengths, 9 heights, 4 girths, 2 widths and 3 angles. The results of this study were follows; First, five factors were extracted by factor analysis. They were foot length, foot girth and width, lateral foot height, medial foot height, and toe flexure factors. Second, four foot types were classified by cluster analysis. Cluster 1 (n=55, 27.4%) referred to the wide and float foot with a long foot length and low heights, and have a little prominent metatarsale fibulare. Cluster 2 (n=59, S.D.=29.3%) represented the small foot with the shortest foot length, the smallest foot girth, and gentle curves on the forefoot. Cluster 3 (n=41, S.D.=20.4%) represented thick foot with open toes compared to the other types. Cluster 4 (M=46, S.D.=22.9%) explained the slant foot to the lateral side that has a flat sole and a little prominent metatarsale tibiale. The findings of this study show that female high school girls in South Korea have diverse shapes and measurements of the foot. The results of this study may be applied for making shoes with the proper fitness for female South Korean youths.
